# The influence of corneal density and thickness on tonometry measurement with goldmann applanation, non-contact and iCare tonometry methods

**DOI:** 10.1007/s10792-022-02216-6

**Published:** 2022-01-13

**Authors:** Ahmed Lubbad, Irene Oluwatoba-Popoola, Melanie Haar, Carsten Framme, Anna Bajor

**Affiliations:** 1 Clinic for Ophthalmology, Hannover Medical School, Hannover, Germany; 2 Carl-Neuberg-Str. 1, 30625 Hannover, Germany

**Keywords:** Intraocular pressure, Tonometry, Corneal densitometry, Central corneal thickness, Glaucoma

## Abstract

**Purpose:**

To evaluate the effect of corneal density and thickness on the accuracy of tonometry readings obtained via three most used techniques.

**Method:**

Intraocular pressures of 45 patients’ right eyes were measured using Goldmann Applanation, iCare, and non-contact tonometry methods. Corneal parameters were obtained using the Pentacam Camera System. Data obtained were analyzed using Paired *t* Test, Pearson’s correlation coefficient, multiple linear regression analysis, and Bland–Altman plots.

**Results:**

The mean corneal thickness was 545.4 ± 3.93 μm. The mean corneal density of total, stromal, 0–2 mm, and 2–6 mm zones were 27.85 ± 6.23 GSU, 24.61 ± 6.05 GSU, 20.76 ± 2.96 GSU, and 20.81 ± 3.51 GSU respectively. IOP readings had a statistically significant correlation with corneal stromal thickness, as well as with total and stromal density. The stromal density, however, showed higher correlation with the three tonometry methods than did the total density (iCare:  − .482 (0.001) stromal density versus− .464 (0.001) total density, NCT: − .376 (0.011) versus − .353 (0.017), GAT: − .306 (0.041) versus − .296 (0.048)). Statistical differences were found in comparing the iCare readings with GAT (*P* < 0,00) and with NCT (*P* < 0,00), with mean differences of 1.8 mmHg ± 2.6 and 2.0 mmHg ± 2.6 respectively. GAT and NCT measurements showed no statistical difference (*P* > 0.05).

**Conclusion:**

This study shows that both central corneal thickness and stromal density are significant influential factors of reliable IOP readings. It is necessary to consider more corneal biomechanical properties, as well as exercise a high degree of caution in any new attempts towards adjusting an IOP-correction equation.

## Introduction

Glaucoma is a multifactorial disease which is diagnosed by evaluating multiple parameters such as intraocular pressure (IOP), visual fields loss, and thinning of the retinal nerve fiber (RNFL). An accurate measurement of IOP is key not only in detecting Glaucoma but also in evaluating the efficacy of a chosen therapy. It is the only risk factor capable of being medically altered in order to slow or stop the asymptomatic progression of this disease.

Goldman applanation tonometry (GAT) has been accepted as the gold standard method of measuring IOP since its invention in 1957 [1]. GAT obtains IOP readings by applying a different amount of mechanical pressure to flatten a pre-measured corneal surface area. Over the past years, different methods have been used to measure IOP. A new routine clinical examination is the Non-Contact Tonometry (NCT), which uses the general principle of Zeiss of Aerotonometry to obtain measurements without touching the globe of the eye [2]. A relatively new method of rebound tonometry most ideal for children and supine patients developed by Kantiola in a hand-held device called iCare measures IOP by detecting the motion of a magnetized probe in a solenoid. High IOP causes shorter stroke time of the probe. Six readings were taken and an average value was generated automatically. The tonometer has a built-in system to indicate if there is any discrepancy among these 6 readings. Whenever an error sign (bar) appeared, a new set of readings was taken. Measurements were conducted on the central cornea. Several studies have identified the influence of different biomechanical and material corneal properties such as corneal thickness, on these three tonometry methods [3–7].

The cornea is a complex tissue mainly made of extracellular matrix components. The physical and biochemical properties of these components are responsible for maintaining their mechanical structure. Collagen fibers and elastin in this tissue are developed and differentiated in a manner conferring strength, elasticity, and optical transparency. Irregularities in the corneal components bring about changes in its clarity and as a result, in its mechanical strength [8].

Oculus pentacam (Oculus Optikgeräte GmbH, Wetzlar, Germany) is a Scheimpflug principle-based, non-invasive imaging system of the ocular anterior segment. A newly added feature to the standard pentagram software provides corneal densitometry analysis by producing a corneal density map of the backscattered light in different areas of the cornea [9]. Using a blue light source, it captures a series of 25 images from which a densitometry map is generated. A Grayscale unit (GSU) is used to describe the density, ranging from 0 GSU of minimum light scattering to 100 GSU of maximal light scattering. In addition to generating four corneal diameter zones of 0–2 mm, 2–6 mm, 6–10 mm, and 10–12 mm from the central apex, it divides the cornea into three layers; anterior, central, and posterior which represent the epithelium, stroma, and endothelium respectively.

In this study, we evaluate the influence of total, stromal (center layer), 0–2 mm, and 2–6 mm corneal density zones, as well as central corneal thickness in the accuracy of IOP measurements using three different tonometry methods.

## Method

This prospective, observational study was carried out at the Medical School Hannover (MHH) in keeping with the tenets of the 1964 Helsinki Declaration, after gaining approval from the local ethics committee.

A total of 45 patients with glaucoma, ocular hypertension or observable papillary excavation, who underwent a hospital-based day-and-night profile of IOP measurements in our clinic were included. Subjects with corneal disease, degenerations or dystrophies, previous ocular surgery or trauma, ocular inflammation were excluded from the study.

After obtaining informed consent, measurement procedures with the Oculus Pentacam (Oculus Optikgeräte GmbH, Wetzlar, Germany) were carried out prior to IOP measurements, to acquire the values of central corneal thickness (CCT) and corneal density. The tonometry values were obtained using the already calibrated tonometers NCT (CT-800, Topcon, Tokyo, Japan), iCare (iCare PRO, Tiolat Oy, Helsinki, Finland), and GAT (Haag Streit AG, Bern, Switzerland) respectively. GAT and iCare measurements were performed by the same doctor (A.L), while NCT was done by an ancillary staff. The average of three NCT values for each eye was used for the study. A drop each of Thilorbin® (Oxybuprocaine Hydrochloride 0,4% and Fluorescein Sodium 0,8%) eye drops was instilled in the eyes before carrying out GAT. GAT was performed last in the sequence of examinations to avoid induced changes in pentagram images and subsequent IOP readings. GAT readings were just obtained once to avoid a corneal-compression-induced aqueous outflow increase that would have affected subsequent IOP readings.

Statistical Package for the Social Sciences (IBM SPSS® Chicago, IL, USA) was used to perform the statistical analysis. In the Intraclass correlation coefficient (ICC) test of pachymetry and densitometry values were highly correlated. Because Intraclass Correlation was high (≥ 0.9 for all parameters except for density zone 0–2 mm 0.864), we analysed the data from right eyes only, to avoid artificially reduced standard deviations. Paired samples t-Test was performed to compare the means of the three tonometry methods. The relationship between CCT and density and the three IOP readings were evaluated by Pearson’s correlation coefficient. Multiple linear regression analysis was performed for the IOP measurements, corneal thickness, and density. In addition, Bland–Altman plots were performed to observe the agreement in IOP readings among the three tonometry methods (Tables 1, 2).

## Results

**Fig. 1:**
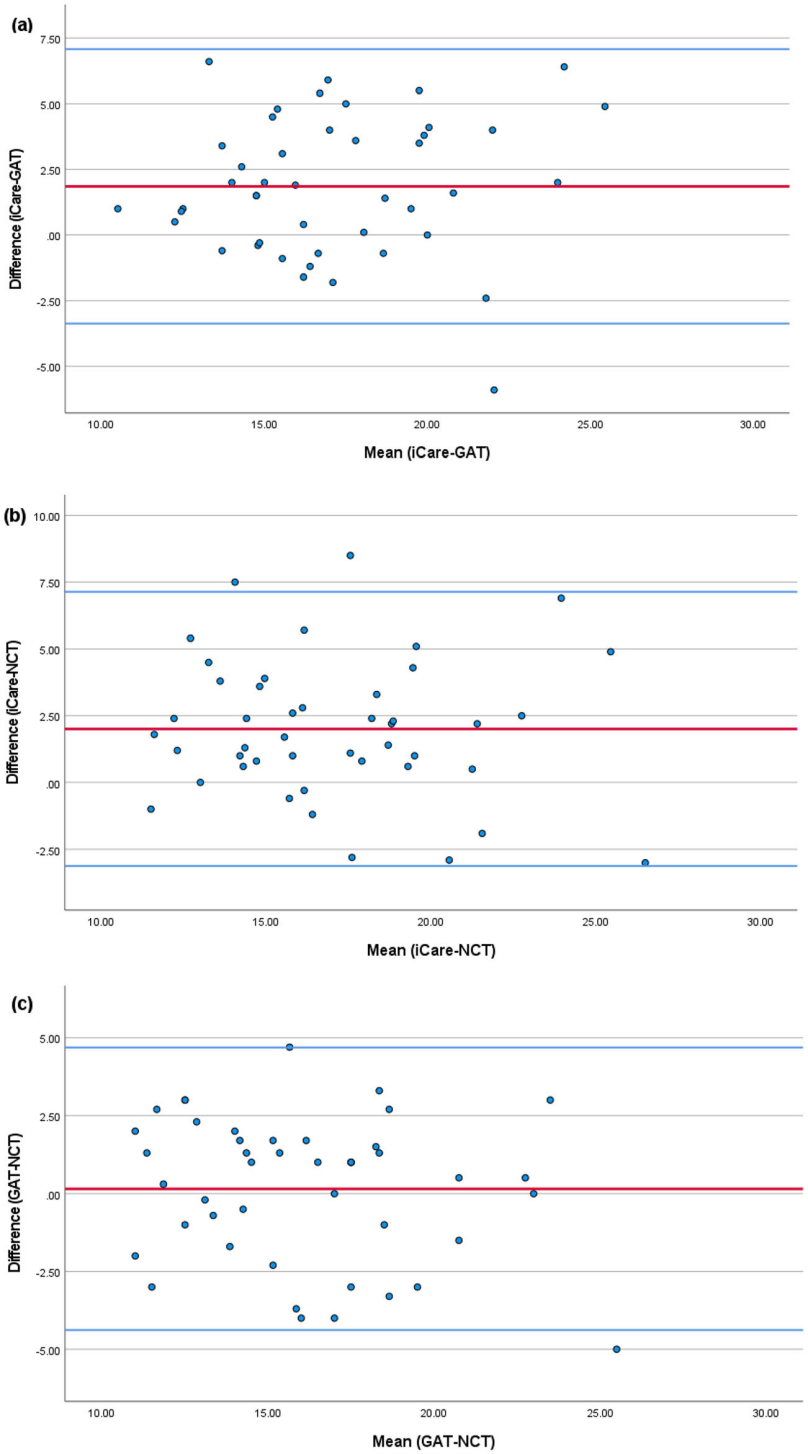
Bland–Altman plots that show differences between **a** iCare-GAT, **b** iCare-NCT, and **c** GAT-NCT tonometry measurements. Statistical differences were found in comparing the iCare-GAT readings with both GAT and NCT with a mean difference of (**a**) 1.8 mmHg ± 2.6 and (**b**) 2.0 mmHg ± 2.6, respectively (*P* < 0.05). There was no statistical difference between GAT and NCT measurements (**c**) 0.15 ± 2.3, *P* > 0.05. The middle line indicates mean difference (bias); upper and lower lines, 95%of limits of agreement

Our study included 45 right eyes of patients with or without glaucoma with a mean age of 61.62 ± 17.64 (median 65). There was no statistical difference between measurements obtained using GAT and NCT procedures (*P* > 0.05). Statistical differences were found in comparing the iCare readings with GAT, with a mean difference of 1.8 mmHg ± 2.6, and with NCT, with a mean difference of 2.0 mmHg ± 2.6. All three methods were however highly correlated (Fig. 1, Table 2).

**Table 1:** Differences and correlations among IOP readings

	Paired samples test	Differences ± SD	r^*p*^ (P Value)
iCare-GAT	P < 0.05	1.8 mmHg ± 2.6	0.736 (< 0.05)
iCare-NCT	P < 0.05	2.0 mmHg ± 2.6	0.77 (< 0.05)
GAT-NCT	P = 0.659023	0.15 mmHg ± 2 .3	0.813 (< 0.05)

**Table 2:** Pearson correlation between IOP readings to CCT and density values (*p* values)

	CCT	Total density	Stromal density	0–2 mm zone	2–6 mm zone
iCare	.479 (0.001)	− .464 (0.001)	− .482 (0.001)	− .109(0.475)	− .290(0.053)
NCT	.484 (0.001)	− .353 (0.017)	− .376 (0.011)	− .203(0.181)	− .228(0.133)
GAT	.329 (0.027)	− .296(0.048)	− .306 (0.041)	− .060(0.693)	− .159(0.295)
CCT	−	− 0.35(0.017)	− .408 (0.005)	− .267(0.077)	− .310(0.038)

**Fig. 2:**
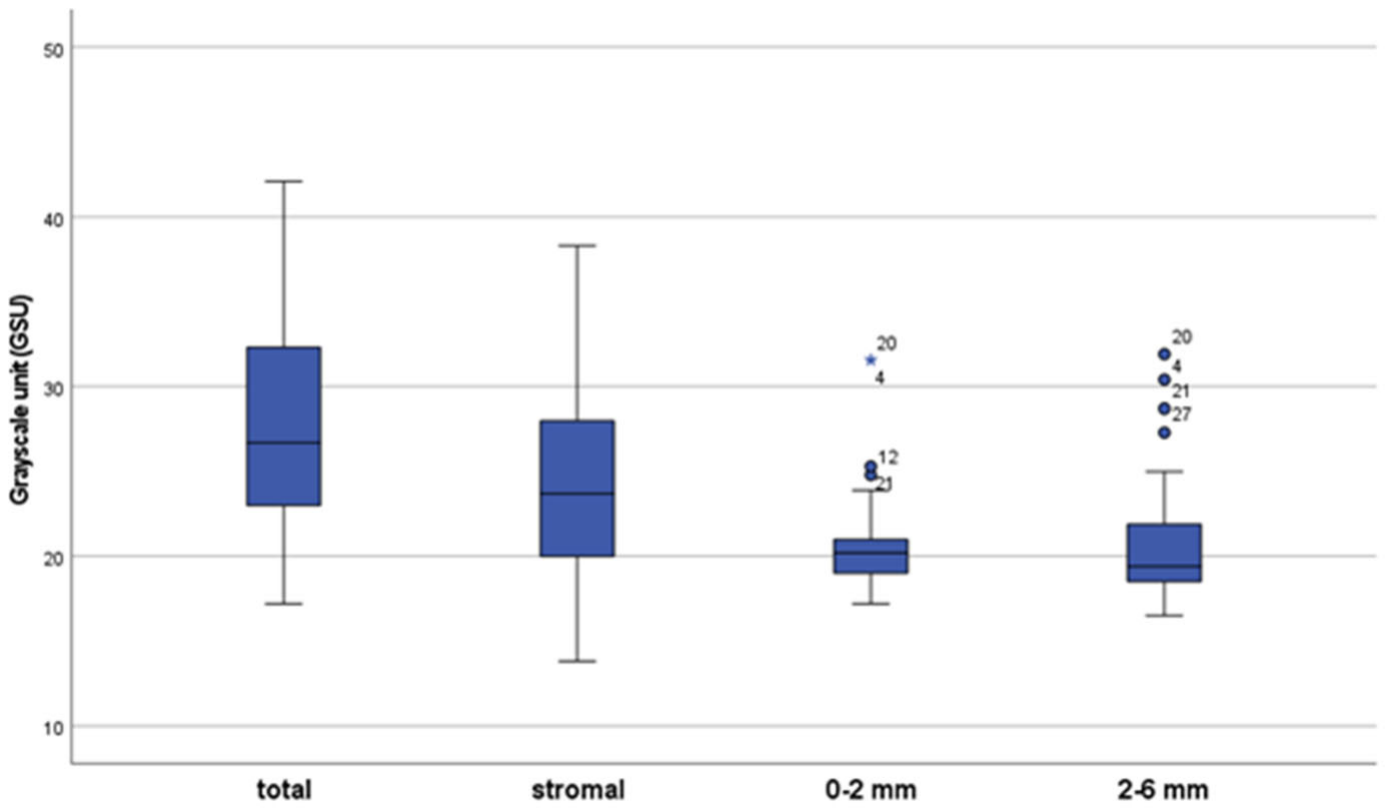
Box plot of corneal densitometry measurements. The mean corneal density of total, stromal, 0–2 mm and 2–6 mm zones were 27.85 ± 6.23 GSU, 24.61 ± 6.05 GSU, 20.76 ± 2.96 GSU, and 20.81 ± 3.51 GSU respectively

**Fig. 3:**
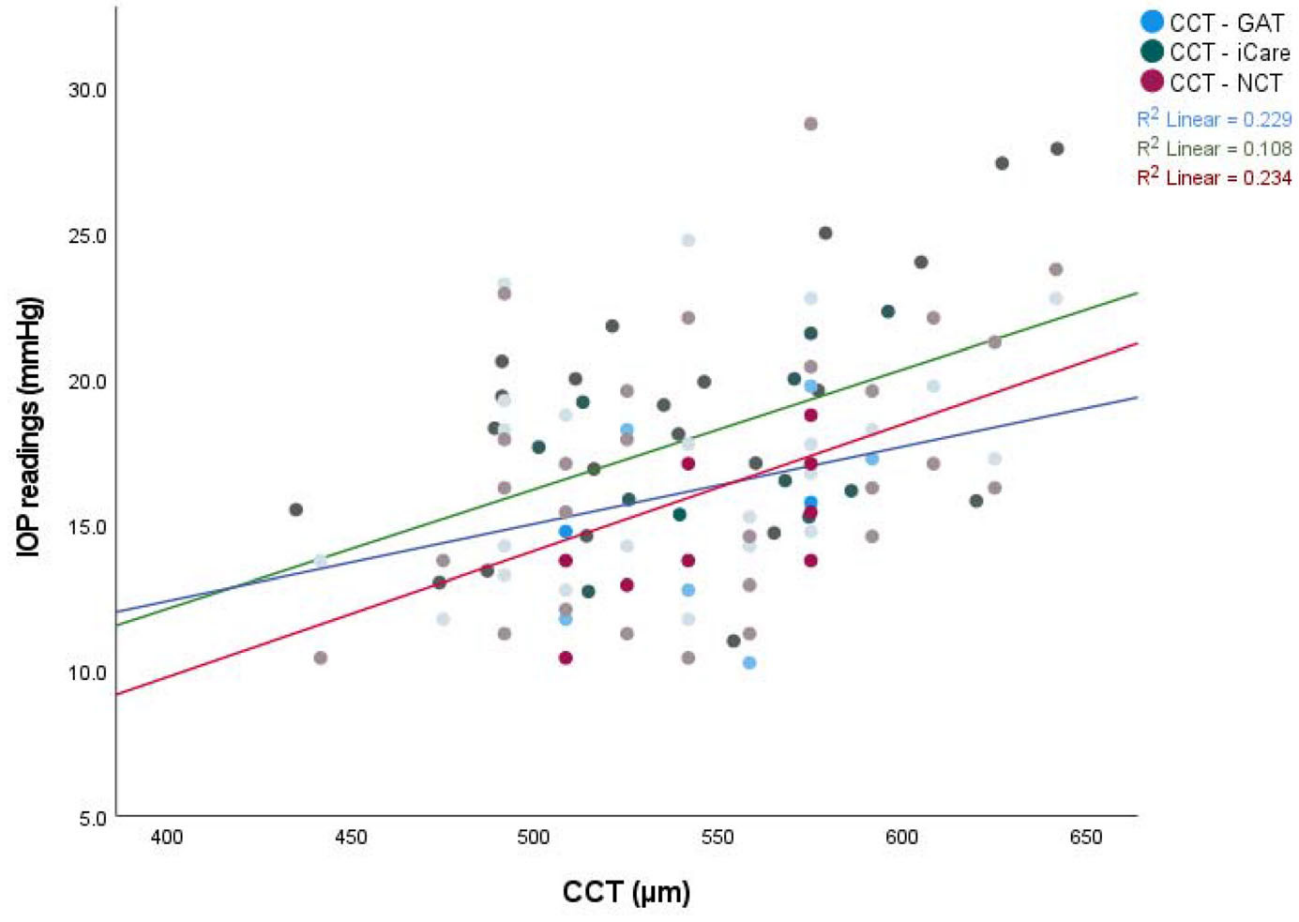
Correlation among IOP readings obtained by GAT in blue (0.329, 0.027), iCare in green (0.479,0.001), and NCT in red solid lines (0.484, 0.001) to CCT were statistically significant of r Pearson and P-Value respectively

**Fig. 4:**
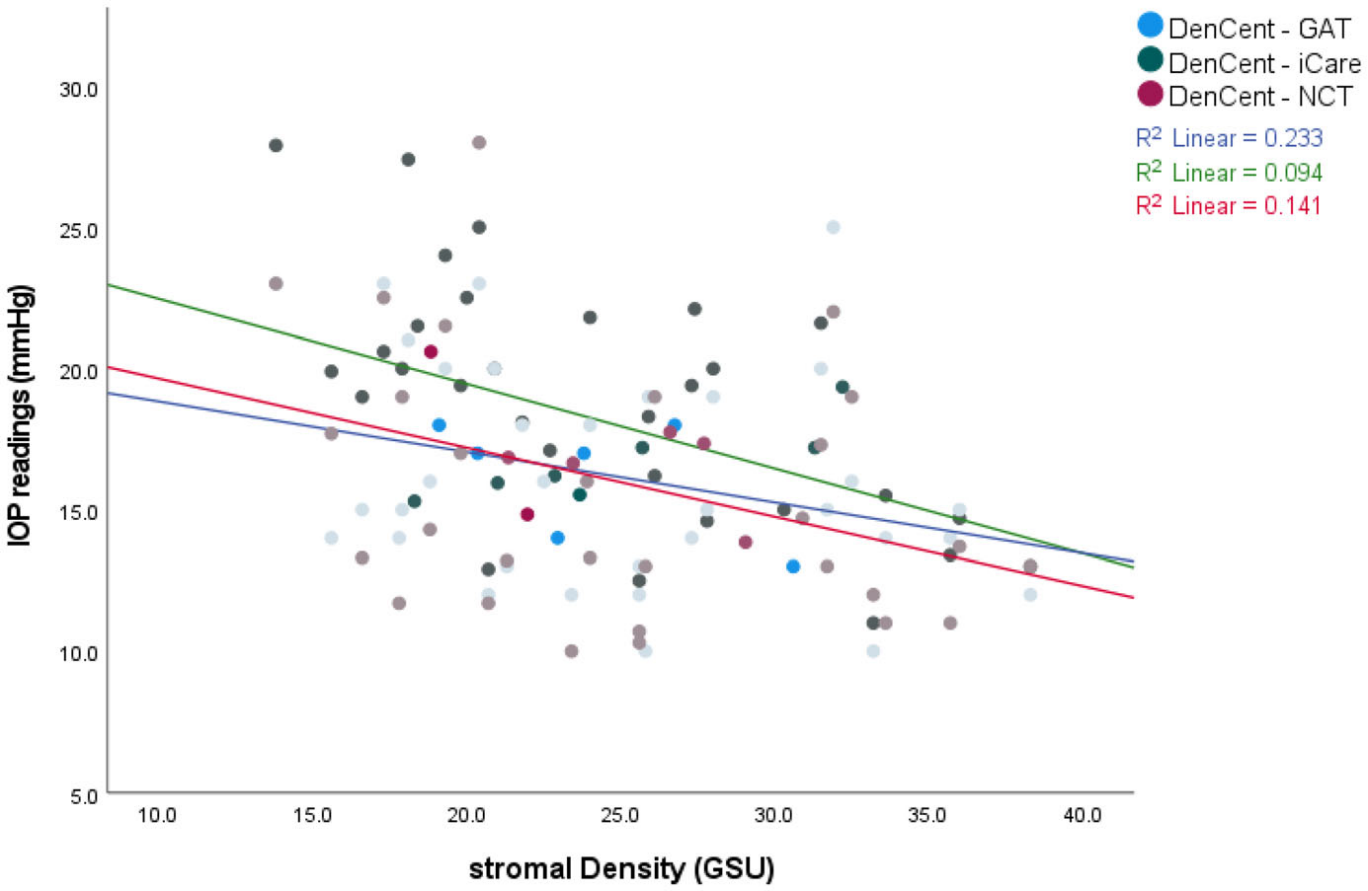
IOP readings obtained by GAT in blue ( − 0.306, 0.041), iCare in green (− 0.482,0.001), and NCT in red solid lines (− 0.376, 0.011) showed a statistically significant correlation to stromal density of r Pearson and *P* Value respectively

We found no effect of gender and age on IOP or CCT readings. Age though significantly affected the density values. Total and stromal density were highly correlated with age (r^p^ 0.761 and 0.754 respectively, *P* < 0.01). The density zones of 0–2 mm and 2–6 mm were less correlated with age (r^p^ 0.424, 0.572 respectively). This correlation was statistically significant (*P* < 0.01). The mean corneal thickness was found to be 545.4 ± 3.93 μm (median 542 μm). The mean corneal density of total, stromal, 0–2 mm, and 2–6 mm zones were 27.85 ± 6.23 GSU, 24.61 ± 6.05 GSU, 20.76 ± 2.96 GSU, and 20.81 ± 3.51 GSU respectively (Fig. 2). There was a statistically significant correlation between IOP readings and CCT, total and stromal density (Figs. 3 and 4). Both 0–2 mm and 2–6 mm density zones were not significantly correlated to any tonometry method. Stromal density showed higher correlation with the three tonometry methods than did the total density.

**Fig. 5:**
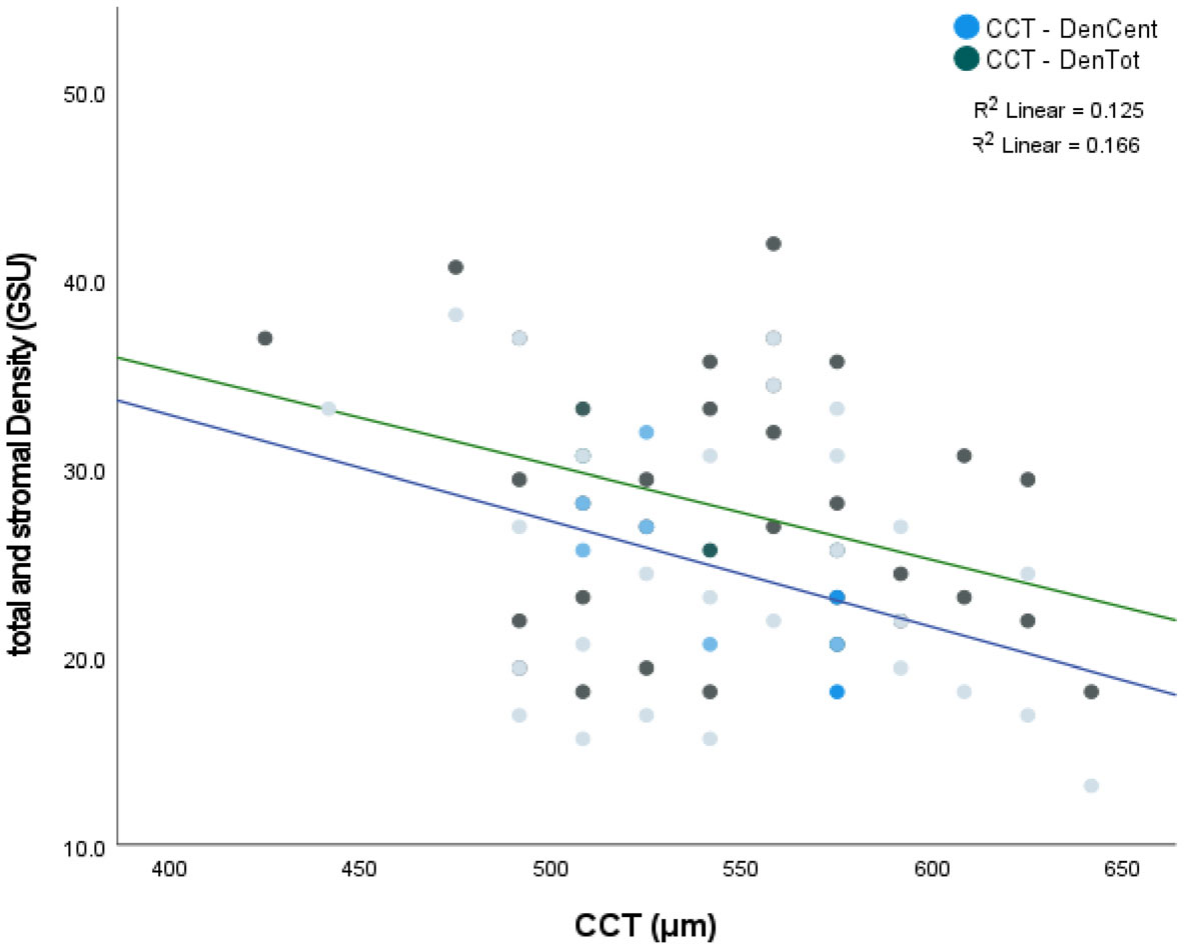
CCT values were negatively correlated to both total in green (− 0.354, 0.17) and stromal density in blue solid lines (− 0.408, 0.005) of r Pearson and *P* Value respectively

CCT showed a significant negative correlation with the total, stromal, and the 2–6 mm density zones, but not with the 0–2 mm zones (Fig. 5). All Pearson and p values are represented in Table 2. Because of high correlations among the density values of total, stromal, zone 0–2, and zone 2–6 mm, the stromal density alone was used in multiple linear regression analysis to avoid multicollinearity.

The linear regression analysis showed significant correlations between IOP readings & CCT, as well as between IOP readings & stromal density. A multiple regression analysis accepted both CCT and stromal density as predictors in ANOVA-Test for Regression (*p* value 0.038 for GAT, < 0.001 for iCare and NCT). Significant correlations were found in the coefficients-test between iCare and CCT (*P* value 0.019, partial correlation 0.353) and stromal density (*P* value 0.017, partial correlation  − 0.358). NCT readings showed a significant correlation with CCT (*P *value 0.009, partial correlation 0.39), but were not significantly correlated to stromal density (*P* value 0.144, partial correlation  − 0.224). Finally, in the GAT readings, a multiple regression analysis rejected both CCT and stromal density (*P* values 0.125 and 0.195), with a partial correlation of 0.235 and  − 0.199 respectively.

## Discussion

IOP evaluation is one of the most important clinical examinations in Ophthalmology, which can most accurately be measured by an invasive manometry. This led to the development of different measurement systems during the last century. In this study, iCare readings were significantly higher than the IOP readings obtained by GAT and NCT. In accordance with other studies, our results confirm overestimation of IOP in iCare readings when compared to GAT [10–13]. Fernandes et al*.* showed a difference of 1.34 mmHg on average in the first study aimed at evaluating the accuracy of iCare readings [11]. Chui et al. showed that other factors such as corneal hysteresis and corneal resistance factor are highly correlated with iCare [6]. Thus various studies show different IOP measurements obtained by NCT and GAT. Tonnu et al. showed that at lower IOP readings, NCT significantly underestimated GAT measurements and overestimated them at higher IOP readings [14]. Jose M. Martinez-de-la-Casa et al. showed a strong agreement between NCT and GAT readings in their study with a mean difference of -0.1 mmHg [15]. Pagoulatos et al. showed higher GAT than NCT measurements in normal, as well as in vitrectomized eyes with silicone oil endotamponade with mean differences of 0.09 mmHg and 3.34 mmHg respectively [16]. The correlation of IOP measurements of NCT and GAT methods seems to be strongly dependent on different factors such as the impact of heart rate, scleral rigidity, as well as the sample size of each study. This reveals that tonometry readings do not always reflect true IOP values but they are clinically similar (within ± 2 mm Hg). It has been confirmed to be affected by corneal resistance brought about by variations in corneal biomechanical factors such corneal thickness, curvature, or hysteresis [5, 6, 17, 18].

The present study confirmed the significant correlation between 3 tonometry methods and CCT. Several studies showed several correction factors depended on this association, resulting in a wide range IOP-correction from 0.12 to 0.7 mm Hg/10 mm corneal thickness [5, 8, 17–19]. Using a cornea biomechanical model, Liu and Roberts showed that the corneal rigidity might have a high influence on IOP measurement and that a higher correction factor is needed by increasing Young’s modulus [20]. In our measurements, we found a negative correlation between the central corneal thickness and the full-thickness corneal density. This result agreed with that of Patel et al*.,* who used confocal microscopy images in vivo for the measurement of corneal thickness and keratocyte density [21].

Elsheikh et al*.* demonstrated an age-associated increase in corneal stiffness [22]. They suggested that this could be related to the increase in the age-related non-enzymatic cross-linking between corneal fibrils and is expected to lead to errors in IOP measurement. This age-related tissue stiffness was inducted in an IOP-correction Eq. 7 Based on this finding, Spoerl et al*.* studied the factor age in the populations of different studies and suggested a new equation adding an age-dependent correction factor in 2012 [23]. In a study with 794 eyes which aimed to describe the normative values of corneal Scheimpflug densitometry, Dhubhghaill et al. showed a significant correlation between age and corneal density [9]. In their study, age was correlated with the total, central layer and 2–6 mm zone with Pearson coefficients of 0.560, 0.484, 0.224 respectively (*P* < 0.001), although not with the 0–2 mm zone. High correlations of age with the total and stromal density (Pearson coefficients > 0.75) were found in our study. We believe this could explain the age-related increase in corneal rigidity found in previous studies.

In this study, the multiple regression analysis showed that CCT and the stromal density are significant influential factors of reliable IOP readings obtained using the three tonometry methods, but the stromal density showed significant coefficient for the iCare readings only. It demonstrates that corneas with high density could lead to an underestimation while corneas with low density to an overestimation of IOP readings. This effect was quite more obvious in rebound tonometry (iCare) than Non-contact or Applanation tonometry methods, which provides an explanation for the differences in IOP-correction equations in the previously mentioned studies. This emphasizes the already postulated idea that more corneal biomechanical properties ought to be considered, and a high degree of caution exercised, in any new attempts towards adjusting an IOP-correction equation. These have to be studied in vitro, proved in vivo, and should not treat the cornea simply as a layer of different cells. Funding.

No funding was received to assist with the preparation of this manuscript.
